# Fellow-Eye Comparison between Phaco-Tanito Microhook Trabeculotomy and Phaco-iStent Inject W

**DOI:** 10.3390/jcm12227005

**Published:** 2023-11-09

**Authors:** Akiko Harano, Ayaka Shimada, Sho Ichioka, Kazunobu Sugihara, Masaki Tanito

**Affiliations:** Department of Ophthalmology, Shimane University Faculty of Medicine, Izumo 693-8501, Japan; ishidaki@med.shimane-u.ac.jp (A.H.); a_shimada2@med.shimane-u.ac.jp (A.S.); sho-ichi.1002@med.shimane-u.ac.jp (S.I.); ksugi@med.shimane-u.ac.jp (K.S.)

**Keywords:** microhook ab-interno trabeculotomy, Tanito microhook (TMH), iStent inject W, intraocular pressure, minimally invasive glaucoma surgery, cataract surgery, fellow-eye comparison

## Abstract

This study aims to compare the surgical efficacy and safety of the Tanito microhook trabeculotomy (TMH-CE) and iStent inject W (Inject-CE) when performed in combination with cataract surgery on the eyes of glaucoma patients. A total of 78 glaucomatous eyes from 39 participants were retrospectively analyzed. Intraocular pressure (IOP), the number of antiglaucoma medications, best-corrected visual acuity (BCVA), anterior chamber flare (ACF), and corneal endothelial cell density (CECD) were all evaluated preoperatively and at multiple postoperative time points. The preoperative IOP was significantly higher in the TMH-CE (19.6 ± 6.7 mmHg) than in the Inject-CE (15.7 ± 3.8 mmHg) (*p* < 0.0001). At the 12-month follow-up, reductions in IOP and the number of medications were more pronounced in the TMH-CE (6.6 mmHg, 27.6% and −1.1, respectively) group than Inject-CE (2.7 mmHg, 12.4% and −0.7, respectively) (*p* < 0.0001 and *p* = 0.0034), while the IOP and medication-number levels were identical between TMH-CE (13.0 ± 3.3 mmHg and 1.3 ± 0.9, respectively) and Inject-CE (12.9 ± 2.6 mmHg and 1.9 ± 0.9, respectively) (*p* = 0.88 and *p* > 0.99, respectively). The TMH-CE group exhibited a higher ACF, a higher frequency of layered hyphema, and a greater anterior chamber floating red blood cells score in the early postoperative periods. Despite these differences, the changes in BCVA, ACF, and CECD were equivalent between the two groups in later follow-up periods. TMH-CE provides a more significant IOP reduction and medication-number reduction compared to Inject-CE, while Inject-CE shows quicker BCVA recovery. This study provides valuable insights for ophthalmologists choosing the most suitable surgical approach for glaucoma and cataract patients.

## 1. Introduction

Glaucoma, a chronic eye condition marked by increased intraocular pressure (IOP), presents a considerable challenge in the field of ophthalmology. The effective management of glaucoma requires interventions that not only lower IOP but also reduce the burden of topical antiglaucoma medications [1]. Among the innovative approaches to treatment, the Minimally Invasive Glaucoma Surgery (MIGS) has emerged as a valuable strategy, offering the promise of favorable outcomes and enhanced safety [2].

In the field of MIGS, the Tanito microhook ab interno trabeculotomy (TMH) procedure, involving the use of a metal-hook device to incise the trabecular meshwork, has gained recognition for its effectiveness in reducing IOP and decreasing the need for antiglaucoma medications [3]. Another notable advancement in the field of MIGS is the iStent, which bypasses the trabecular meshwork by implanting a small stent [4].

Currently, the iStent is available in two generations: the first-generation iStent and second-generation iStent Inject W. Previously, multiple studies were conducted to compare the effectiveness and safety of MIGS procedures between Kahook Dual Blade (KDB) and the first- [5,6,7,8] or second-generation [9] iStent. In our previous report, fellow-eye comparison showed that the IOP reduction was greater with TMH than with the first-generation iStent when combined with cataract surgery [10]. The second-generation iStent is the most compact trabecular device currently on the market, occupying less than 0.5 mm [11]. Its design streamlines surgical technique and enables the concurrent implantation of two stents, with the goal of achieving greater IOP reduction compared to the first-generation iStent [11].

More recently, surgical effectiveness has been compared between the TMH and iStent Inject W in a retrospective study conducted in Japan [12]. To the authors’ knowledge, there is no study that has compared the TMH and second-generation iStent in a same patient. Here, we present a comparative analysis of effectiveness and complications between patients who underwent TMH combined with cataract surgery in one eye and those who received an Inject W implantation combined with cataract surgery in the fellow eye.

## 2. Subjects and Methods

### 2.1. Participants

This study adhered to the tenets of the Declaration of Helsinki. The institutional review board (IRB) of Shimane University Hospital reviewed and approved the research (IRB No. 20200517-1, with a revised protocol issued on 15 June 2023). Prior to surgery, all participants supplied written informed consent for surgery. It is important to note that IRB approval did not mandate individual written informed consent for publication from each patient. Instead, the study protocol was made available at the study institutions to inform participants about the research. Only de-identified data were utilized in the statistical analyses.

We conducted a retrospective analysis, including participants who underwent surgeries performed by two surgeons (M.T. and K.S.) at Shimane University Hospital between October 2020 and March 2022. The inclusion criteria included individuals who underwent, within one week, a TMH combined with cataract surgery in one eye (TMH-CE group) and an iStent Inject W implantation combined with cataract surgery in the fellow eye (Inject-CE group). Additionally, eligible participants had no prior history of intraocular or glaucoma surgeries. They had recorded preoperative Goldmann applanation tonometry-measured IOPs and the number of antiglaucoma medications at 2 weeks (1–3 weeks), 3 months (2–4 months), 6 months (5–7 months), 9 months (8–10 months), and 12 months (11–14 months). The surgeons chose the TMH for eyes with severe visual disturbance in almost all cases (in only one case, the iStent inject W was implanted for an eye with more severe visual-field defect). The exclusion criteria included intraoperative complications, including posterior capsular rupture, Zinn’s zonular dialysis, and goniodialysis. As a result, 78 eyes in 39 of the participants (mean age ± standard deviation (SD), 73.0 ± 7.4 years; 18 men, 21 women) who fulfilled the inclusion and exclusion criteria in the database were subjected to this study.

We collected the following data through a medical chart review: age; gender; eye laterality; glaucoma type (including primary open-angle glaucoma (POAG), exfoliation glaucoma (EXG), and other forms of glaucoma); IOP; number of medications; best-corrected visual acuity (BCVA); anterior chamber flare (ACF) measurements obtained using the FM-600 laser flare meter (Kowa, Nagoya, Japan); corneal endothelial cell density (CECD) determined with the EM-3000 specular microscope (Tomey, Nagoya, Japan); visual field mean deviation (MD) assessed by the Central 30-2 Program on the Humphrey Visual Field Analyzer (Carl Zeiss Meditec, Dublin, CA, USA); and recorded surgical complications. In cases where exfoliation material deposition was observed in only one eye during slit-lamp examination, the individual was classified as having unilateral EXG, and the other eye was categorized as having POAG.

The severity of each hyphema was estimated and given an R score according to the Shimane University Hyphema Scoring System (SU-R) [13]. This system, which is specifically designed to classify postoperative hyphema, categorizes the floating red blood cells (RBCs) in AC from 0 to 3. Here, 0 indicates there are no floating RBCs in the AC; 1 indicates that floating RBCs can be seen but iris patterns are observed clearly in the entire AC; 2 indicates that floating RBCs can be seen but unclear iris patterns are observed; 3 indicates that dense floating RBCs are seen and iris patterns are not observed.

### 2.2. Surgical Procedures

Prior to the TMH or iStent Inject W procedures, all patients underwent phacoemulsification cataract surgery. This surgery involved creating a 2.2 mm-wide clear corneal incision at the 9–10 o’clock position. For the right eye, this incision was made temporally; for the left, it was created nasally. Subsequently, a one-piece soft acrylic intraocular lens was placed into the capsular bag through the same clear corneal incision.

For cases that underwent the TMH, we utilized spatula-shaped microhooks (M-2215S, 2215R, and 2215L, Inami, Tokyo, Japan) designed specifically for this procedure. To facilitate the surgery, viscoelastic material (1% sodium hyaluronate, Provisc, Alcon Japan, Tokyo, Japan, or Opegan Hi, Santen Pharmaceutical, Osaka, Japan) was introduced into the AC via clear corneal incisions made with a 20-gauge microvitreoretinal knife (Mani, Utsunomiya, Japan) at the 2–3 o’clock and 9–10 o’clock positions. A microhook was then inserted into the AC through the corneal incision, and the angle opposite to the corneal port was visualized using a Swan–Jacob gonioprism lens (Ocular Instruments, Bellevue, WA, USA). The microhook tip was carefully advanced into Schlemm’s canal and used to circumferentially incise the inner wall of Schlemm’s canal and the trabecular meshwork. This incision encompassed more than 3 clock hours, primarily at the nasal position (30 eyes, 77.0%), or both the nasal and temporal positions (9 eyes, 23.0%). In cases that underwent the iStent inject W implantation (Glaukos Corporation, San Clemente, CA, USA), the two stents were inserted into Schlemm’s canal through the trabecular meshwork under observation using a Swan–Jacob gonioprism lens. Following the TMH or iStent Inject W procedures, we removed the viscoelastic material and closed the corneal ports through stromal hydration. Subsequently, at the conclusion of the surgery, we administered a subconjunctival injection of a steroid (2 mg betamethasone sodium phosphate, Rinderone, Shionogi Pharmaceutical) and applied 0.3%-ofloxacin ointment (Tarivid, Santen Pharmaceutical). Additionally, we prescribed topical 1.5%-levofloxacin (Nipro, Osaka, Japan) and 0.1%-betamethasone (Sanbetason, Santen Pharmaceutical) to be used four times daily for 3– 4 weeks postoperatively for all patients.

### 2.3. Statistical Analysis

The sample size of this study (*n* = 78, with 39 eyes in each procedure group) provided over 99% power to detect an average difference in postoperative IOP reduction of 4.4 mmHg between eyes (7.1 ± 6.6 mmHg in eyes that underwent TMH versus 2.7 ± 4.0 mmHg in eyes implanted with the inject W) at the 12-month postoperative point. Power calculations were based on a type-I error of 0.05 and a two-sided test.

To address potential biases stemming from the inclusion of both eyes from the same patient, we conducted comparisons of IOP reductions and changes in the number of antiglaucoma medications using mixed-effects regression models. In this analysis, each patient’s identification number was treated as a random effect, and the time period and glaucoma surgical procedure were regarded as fixed effects. For inter-group comparisons at each observation period, we employed the Wilcoxon signed-rank test for continuous data and Fisher’s exact probability test for categorical data.

We analyzed the estimated survival probability for achieving qualified IOP control using Kaplan–Meier curves. Successful IOP control was evaluated through survival curve analysis, classifying cases as uncensored under the following conditions: when the IOP exceeded 15 mmHg (criterion A) or 12 mmHg (criterion B) after 3 months postoperative; when the IOP reduction was less than 20% after 3 months postoperative (both definitions); when additional glaucoma surgery was performed at any time (both definitions); or when there was a loss of light perception at any time (both definitions). All other cases were regarded as censored. The use or non-use of antiglaucoma medication was not considered in the survival curve analysis because the majority of current cases continued medication postoperatively. We used log-rank tests to assess differences in survival rates between surgical groups.

All statistical analyses were two-sided, and *p* = 0.05 was considered statistically significant. The data are presented as means ± SD for continuous variables and in numbers and percentages for categorical variables. To conduct the statistical analyses, decimal BCVA records were converted to the logarithm of the minimum angle of resolution (LogMAR). Counting fingers, hand motions, light perception, and no light perception were converted to decimal VAs of 0.0025, 0.002, 0.0016, and 0.0013, respectively [14]. All statistical analyses were performed using JMP Pro statistical software version 14.2 (SAS Institute, Inc., Cary, NC, USA).

## 3. Results

Table 1 summarizes the demographic data of the participants, which include their age, gender, laterality, preoperative MD values, and glaucoma types. The glaucoma types represented in this study were primarily POAG (66.6%), followed by EXG (25.6%) and other glaucoma types (8.0%). Notably, there was a significant difference in the distribution of glaucoma subtypes between the two groups (*p* = 0.0046), with the TMH-CE group having a higher proportion of EXG cases compared to the Inject-CE group. The eyes that underwent the TMH also had significantly (*p* < 0.0001) greater severe visual field defects.

Table 2 shows the comparison of the IOPs and numbers of antiglaucoma medications preoperatively and postoperatively between the TMH-CE and Inject-CE groups. Preoperatively, IOP was significantly higher (*p* < 0.0001) and the number of antiglaucoma medication (*p* = 0.0063) was significantly more in the TMH-CE group than in the Inject-CE group. During the study periods, IOP was significantly lower in Inject-CE than TMH-CE at 1 month (*p* = 0.012), while the IOP in other postoperative follow-up periods, and the number of medications during all postoperative follow-up periods, were identical between the groups for up to 12 months postoperative.

Table 3 presents postoperative reductions in IOP (ΔIOP) and the number of antiglaucoma medications (Δmedication) for both the TMH-CE and Inject-CE groups. With the exception of the 3-month postoperative period, the TMH-CE group had significantly greater reductions in ΔIOP compared to the Inject-CE group. Similarly, except for the 1-month and 3-month postoperative periods, the Δmedication was significantly greater in the TMH-CE group compared to the Inject-CE group.

**Figure 1:**
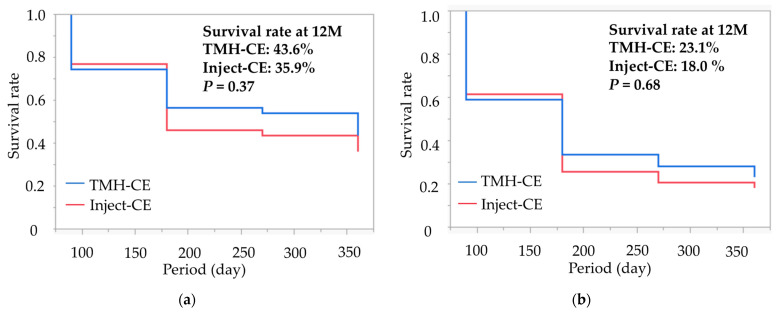
Kaplan–Meier survival curves for successful intraocular pressure (IOP) control in the iStent Inject W (Inject-CE) and microhook ab interno trabeculotomy (TMH-CE) groups. Two failure criteria, criterion A (**a**) and criterion B (**b**), were used: IOP < 20% reduction from preoperative IOP value and/or >15 mmHg for criterion A, and IOP < 20% reduction from preoperative value and/or >12 mmHg for criterion B. Patients who did not meet these IOP-failure criteria, as well as those requiring additional glaucoma surgery and/or experiencing no light perception, were considered failures. The log-rank statistics for the comparison between the two groups yielded *p*-values of 0.37 and 0.68, respectively.

Kaplan–Meier survival curves for successful IOP control in both groups are displayed in Figure 1. The cumulative survival rates for the TMH-CE and Inject-CE groups at 12 months were 43.6% and 35.9% for criterion A, and 23.1% and 18.0% for criterion B, respectively. The log-rank statistics between the two groups for these criteria were *p* = 0.54 and *p* = 0.68, respectively.

Table 4 displays a comparison of postoperative complications and interventions between the TMH-CE and Inject-CE groups. The frequency of layered hyphema was significantly higher in the TMH-CE group compared to the Inject-CE group (*p* = 0.025); however, the frequency of IOP spikes exceeding 30 mmHg, cystoid macular edema (CME) detected by optical coherence tomography (OCT), and fibrin in the anterior chamber were similar between the groups (*p* > 0.99). No additional glaucoma surgeries were required in either group during the follow-up period.

The comparison of the SU-R score is presented in Table 5, showing that the SU-R score in the TMH-CE group was higher than that in the Inject-CE group (*p* = 0.0002).

Table 6 presents comparisons of preoperative and postoperative BCVAs, ACF, and CECD between the TMH-CE and Inject-CE groups. Preoperatively, BCVA was worse and CECD was lower in the TMH-CE group compared to the Inject-CE group, while ACF was similar between both groups. Postoperatively, BCVA remained consistently worse in the TMH-CE group compared to the Inject-CE group during all follow-up periods, except for 3 months postoperative. One month postoperative, ACF was higher in the TMH-CE group than in the Inject-CE group, while ACF was equivalent in other follow-up periods. At 6 months postoperative, CECD was lower in the TMH-CE group than in the Inject-CE group, but it was equivalent between the two groups at 12 months postoperative.

Table 7 displays the postoperative changes in BCVA (ΔBCVA), ACF (ΔACF), and CECD (ΔCECD) compared to the preoperative values. ΔBCVA was significantly smaller in the Inject-CE group than in the TMH-CE group at 2 weeks postoperative but was equivalent between the groups at 1 month postoperative and later, up to 12 months. ΔACF was significantly larger in the TMH-CE group than in the Inject-CE group at 2 weeks and 1 month postoperative but was equivalent between both surgical groups at 3 months postoperative and later, up to 12 months. ΔCECD was equivalent between both surgical groups at 6 and 12 months postoperative.

## 4. Discussion

In the current study, the effectiveness and complications of the TMH and iStent inject W combined with cataract surgery were compared between the eyes of each subject. Several key clinical findings were observed. First, the TMH resulted in a greater reduction in IOP postoperatively compared to the implantation of the iStent inject W. Second, the TMH-CE group had a significantly higher frequency of postoperative layered hyphema and a higher density of floating RBCs in the AC compared to the Inject-CE group. Third, postoperative improvement in BCVA due to cataract surgery became evident earlier in eyes that were implanted with the iStent inject W compared to eyes that underwent the TMH. This study is unique in its approach, as it conducted a fellow-eye comparison to assess IOP reduction and safety profiles between the TMH-CE and Inject-CE, providing valuable insights into the two surgical procedures.

Regarding the first point, we observed larger reductions of IOP and medication number in the TMH-CE group than in the Inject-CE group. Previously, in an intraindividual comparative study over a 12-months follow-up in POAG, Trabectome and the iStent inject W were both effective in lowering IOP without a significant difference between the two groups [15]. On the other hand, KDB seemed to offer an advantageous IOP reduction compared to the first-generation iStent [5,6,7,8] and even compared to the iStent inject W [9,16]. From other groups, equivalence in IOP reduction between TMH and KDB were reported [17,18]. Very recently, no remarkable difference in IOP reduction between TMH and iStent inject W was reported from Japan [12]. Collectively, TMH can be more effective in IOP reduction than the second-generation iStent, although the difference was not large enough to detect consistently among the different settings of the studies. Reductions of IOP during trabeculotomy result from relieving the resistance to aqueous flow by the cleavage of the trabecular meshwork and inner walls of Schlemm’s canal at the point of outflow resistance of the aqueous humor. Similarly, the iStent inject W, which bypasses the trabecular meshwork and causes Schlemm’s canal to dilate, can also effectively lower IOP [19]. The smaller inject-W aperture, with a device that occupies less than 0.5 mm [11], may be more vulnerable to trabecular meshwork reactivity than the TMH, during which a wider incision of the inner wall of Schlemm’s canal is created and can sustain aqueous humor drainage by goniotomy procedures. This might explain why the TMH produced a greater IOP reduction than the iStent inject W in the current study. The reduction of IOP after goniotomy was directly affected by the preoperative IOP level, i.e., larger postoperative IOP reduction was achieved in eyes with higher preoperative IOP [3]. Accordingly, it is important to note that significantly higher preoperative IOP in the TMH-CE group than the Inject-CE group should affect the difference in IOP reduction in the current study.

There was no difference in the cumulative survival rates in the two groups in the current study, while the survival rate in the TMH-CE group was higher than in the first-generation iStent group in our previous study [10]. The discrepancy observed in these studies can be explained by the IOP-lowering effect of the second-generation iStent, which is higher than the first-generation iStent [20,21]. In the previous report, the cumulative survival rates in the TMH and iStent groups at 12 months were 53.1% and 37.5% for IOP control ≤ 15 mmHg and IOP reduction ≥ 20% (i.e., the same as criterion A in this study), and 40.6% and 18.8% for IOP control ≤ 12 mmHg and IOP reduction ≥ 20% (i.e., the same as criterion B in this study), respectively. The success rates for both criteria seemed equivalent between the first-generation iStent in our previous study and the iStent inject W in the current study. However, the success rate of the TMH-CE in the current report might be worse than that in the previous report. Accordingly, the non-significant difference in success rates between the TMH-CE and Inject-CE groups in the current report can be explained by the lower success rate in the TMH-CE group. There are some differences in subjects’ characteristics between our previous report [10] and the current report. The preoperative IOP in the TMH-CE group in this report is higher than in the previous report. Additionally, in the previous report, TMH incision was made at both nasal and temporal quadrants in all cases but, in the current report, incision was made at both the nasal and temporal sides in one-fourth of the subjects but at only the nasal side in three-fourths of the subjects. Previous reports suggested no clinically remarkable efficacy in terms of the difference in goniotomy extent [22,23]. However, the difference in the extent of incision might affect the survival curve analysis in this study. Furthermore, the eyes that underwent the TMH had more EXG than those implanted with the iStent inject W. These situations might make the survival rate of TMH worse.

The second and third observations in our study are closely related. We found that the frequency of early postoperative layered hyphema and the SU-R score were significantly higher in the TMH-CE group compared to the Inject-CE group. In a previous study, we reported higher hyphema scores in eyes that underwent TMH compared to eyes implanted with the first-generation iStent on postoperative days 1, 2, and 3 [13]. Our current study revealed that postoperative hyphema is even less common with the second-generation iStent than with the TMH. This reduced hyphema likely explains the smaller ΔACF in the Inject-CE group compared to the TMH-CE group at 2 weeks and 1 month postoperative. The lower incidence of hyphema and anterior chamber inflammation in the Inject-CE group resulted in the difference in ΔBCVA at 2 weeks postoperative. Therefore, when compared to the TMH, the implantation of the iStent inject W offers the advantage of earlier visual recovery after surgery. Apart from these early postoperative reactions, the rates of complications, ΔBCVA, ΔACF, and ΔCECD did not show significant differences during the study period. Thus, both surgical procedures were equivalent in terms of safety profiles.

The present study had several limitations. It was a retrospective study and lacked randomization. Although the statistical power calculation indicated a 99% power to detect inter-group differences in IOP at 12 months, the relatively small sample size and short observation period were two limitations of this study. Consequently, our results should be confirmed through more rigorous studies, such as randomized trials and multicenter studies, and those with longer observation periods. The inclusion of a population predominantly consisting of elderly individuals may restrict the generalizability of our findings. The implantation of the iStent inject W was carried out in eyes with relatively lower preoperative IOP, a higher prevalence of POAG, and milder visual field impairment, which introduced selection bias. Despite these limitations, our study possessed several strengths, including a fellow-eye comparison to mitigate the influence of patient-specific factors, a sufficiently large sample size for the detection of clinically significant differences across all parameters, and comprehensive assessments of patient clinical characteristics encompassing ACF, CECD, and hyphema score. Given that the TMH can be performed using reusable hooks and does not require costly implants, it appears to be a cost-effective option for patients with glaucoma.

## 5. Conclusions

This single center, retrospective study demonstrated that the amount of IOP reduction as well as the amount of medication number reduction achieved with the TMH were greater than with the iStent inject W when both were combined with cataract surgery; however, the achieved IOP levels were identical between both procedures in the fellow-eye comparison. This study highlights the clinical efficacy of the TMH during cataract surgery for reducing IOP as well as early postoperative recovery of vision after the iStent inject W implantation during cataract surgery.

## Figures and Tables

**Table 1:** Demographic data.

Parameters	TMH-CE	Inject-CE	*p*-Value
Number	39		
Age, years			
Mean ± SD	73.0 ± 7.4		
95% CI	71.4, 74.7		
Gender			
Male, *n* (%)	18 (46)		
Female, *n* (%)	21 (54)		
Glaucoma type			
POAG, *n* (%)	20 (38.5)	32 (61.5)	0.0046 **
EXG, *n* (%)	16 (80.0)	4 (20.0)	
Others, *n* (%)	3 (50.0)	3 (50.0)	
Preoperative LogMAR VA			
Mean ± SD	0.3 ± 0.3	0.2 ± 0.3	0.0011 **
95% CI	0.2, 0.4	0.1, 0.3	
Preoperative IOP, mmHg			
Mean ± SD	19.6 ± 6.7	15.7 ± 3.8	<0.0001 **
95% CI	17.5, 21.8	14.4, 16.9	
Preoerative medication			
Mean ± SD	3.0 ± 1.4	2.6 ± 1.2	0.0063 **
95% CI	2.6, 3.5	2.2, 3.0	
Axial length, mm			
Mean ± SD	24.2 ± 1.6	24.2 ± 1.7	0.72
95% CI	23.7, 24.7	23.7, 24.7	
MD, dB			
Mean ± SD	−13.9 ± 8.4	−6.1 ± 5.9	<0.0001 **
95% CI	−16.6, −11.2	−8.0, −4.2	
Severity of visual field defects			
Mild (MD > −6dB), *n* (%)	7 (23.3)	23 (76.7)	0.0003 **
Moderate (−12 < MD < −6 dB), *n* (%)	13 (56.5)	10 (43.5)	
Severe (MD < −12 dB)	19 (76.0)	6 (24.0)	

*p* values are calculated by the Wilcoxon signed rank test for continuous data and using Fisher’s exact probability test and the Wilcoxon test for categorical data. The ** corresponds to the significance level at 1% (*p* < 0.01) for *t*-test or Fisher’s exact probability test. TMH, Tanito microhook trabeculotomy; CE, cataract extraction; SD, standard deviation; CI, confidence interval; POAG, primary open-angle glaucoma; EXG, exfoliation glaucoma; VA, visual acuity; IOP, intraocular pressure; MD, mean deviation.

**Table 2:** IOP and antiglaucoma medications at preoperative and postoperative visits.

Parameters	IOP, mmHg			Number of Medications, *n*		
	TMH-CE	Inject-CE	*p*-Value	TMH-CE	Inject-CE	*p*-Value
Preoperative value						
Mean ± SD	19.6 ± 6.7	15.7 ± 3.8	<0.0001 **	3.0 ± 1.4	2.6 ± 1.2	0.0063 **
95% CI	17.5, 21.8	14.4, 16.9		2.6, 3.5	2.2, 3.0	
2 weeks postoperatively						
Mean ± SD	14.2 ± 4.3	13.9 ± 3.9	0.67	1.8 ± 0.9	1.7 ± 0.8	0.16
95% CI	12.8, 15.6	12.6, 15.2		1.5, 2.1	1.5, 2.0	
1 month postoperatively						
Mean ± SD	12.1 ± 3.8	12.3 ± 3.4	0.91	1.8 ± 0.9	1.7 ± 0.9	0.08
95% CI	10.8, 13.5	11.1, 13.4		1.5, 2.1	1.4, 2.0	
3 months postoperatively						
Mean ± SD	14.0 ± 3.5	12.8 ± 2.4	0.012 *	1.8 ± 0.9	1.7 ± 0.8	1.00
95% CI	12.3, 15.6	11.6, 13.9		1.4, 2.1	1.3, 2.0	
6 months postoperatively						
Mean ± SD	12.3 ± 2.6	11.8 ± 2.4	0.17	1.8 ± 0.7	1.8 ± 0.7	0.32
95% CI	11.4, 13.2	11.0, 12.6		1.6, 2.1	1.5, 2.0	
9 months postoperatively						
Mean ± SD	12.3 ± 2.9	12.5 ± 2.5	0.34	2.0 ± 0.9	1.9 ± 0.9	0.98
95% CI	11.3, 13.3	11.6, 13.3		1.7, 2.3	1.6, 2.2	
12 months postoperatively						
Mean ± SD	13.0 ± 3.3	12.9 ± 2.6	0.88	1.9 ± 0.9	1.9 ± 0.9	>0.99
95% CI	11.9, 14.1	12.1, 13.8		1.6, 2.2	1.6, 2.2	

*p* values are calculated by the Wilcoxon signed rank test. The * and ** correspond to the significance level at 5% (*p* < 0.05) and 1% (*p* < 0.01) for the Wilcoxon signed rank test. TMH, Tanito microhook trabeculotomy; CE, cataract extraction; SD, standard deviation; CI, confidence interval.

**Table 3:** Postoperative reduction of IOP and antiglaucoma medications.

Parameters	ΔIOP, mmHg			Δmedication, *n*		
	TMH-CE	Inject-CE	*p*-Value	TMH-CE	Inject-CE	*p*-Value
2 weeks postoperatively						
Mean ± SD	−5.4 ± 6.1	−1.8 ± 5.1	0.0006 **	−1.2 ± 1.3	−0.9 ± 1.2	0.0065 **
95% CI	−7.4, −3.4	−3.4, −0.1		−1.7, −0.8	−1.3, −0.5	
1 month postoperatively						
Mean ± SD	−7.6 ± 6.6	−3.7 ± 4.0	<0.0001 **	−1.0 ± 1.4	−1.0 ± 1.2	0.3100
95% CI	−9.9, −5.3	−5.0, −2.3		−1.7, −0.7	−1.4, −0.5	
3 months postoperatively						
Mean ± SD	−6.6 ± 8.4	−3.9 ± 4.7	0.09	−0.8 ± 1.1	−0.7 ± 1.1	0.13
95% CI	−10.4, −2.6	−6.0, −1.7		−1.4, 0.3	−1.2, −0.1	
6 months postoperatively						
Mean ± SD	−7.6 ± 6.6	−3.9 ±3.9	0.0002 **	−1.2 ±1.3	−0.8 ± 1.2	0.0033 **
95% CI	9.8, −5.4	−5.2, −2.6		−1.6, −0.8	−1.2, −0.4	
9 months postoperatively						
Mean ± SD	−7.1 ± 6.6	−2.7 ± 4.0	<0.0001 **	2.0 ± 0.9	1.9 ± 0.9	0.033 *
95% CI	−9.3, −4.8	−4.1, −1.3		1.7, 2.3	1.6, 2.2	
12 months postoperatively						
Mean ± SD	−6.6 ± 7.0	−2.7 ± 4.0	<0.0001 **	−1.1 ± 1.3	−0.7 ± 1.1	0.0034 **
95% CI	−8.9, −4.4	−4.1, −1.4		−1.6, −0.7	−1.1, −0.3	

*p* values are calculated by the Wilcoxon signed rank test. The * and ** correspond to the significance level at 5% (*p* < 0.05) and 1% (*p* < 0.01) for the Wilcoxon signed rank test. ΔIOP indicates reduction in intraocular pressure from preoperative value; Δmedication indicates reduction in the number of medications from preoperative value; TMH, Tanito microhook trabeculotomy; CE, cataract extraction; SD, standard deviation; CI, confidence interval.

**Table 4:** Postoperative complications and interventions.

Parameters	TMH-CE	Inject-CE	*p*-Value
Layered hyphema, *n* (%)	10 (26)	2 (5)	0.025 *
IOP spikes, *n* (%)	3 (7.7)	2 (5)	>0.99
Cystoid macular edema, *n* (%)	0 (0)	1 (2.6)	>0.99
Additional glaucoma surgery, *n* (%)	0 (0)	0 (0)	-
Fibrin in anterior chamber, *n* (%)	2 (5)	1 (2.6)	>0.99

Comparisons between the TMH-CE and iStent groups using Fisher’s exact probability test. IOP spikes are defined as IOP greater than 30 mmHg. The * corresponds to the significance levels at 5% (*p* < 0.05). TMH, Tanito microhook trabeculotomy; CE, cataract extraction; *n*, number of participants; IOP, intraocular pressure.

**Table 5:** Comparison of the rate of SU-R score between TMH-CE and Inject-CE.

SU-R Score	TMH-CE	Inject-CE	*p*-Value
Score 0, *n* (%)	6 (15.0)	6 (15.0)	0.0002 **
Score 1	7 (18.0)	16 (41.0)	
Score 2	10 (25.6)	16 (41.0)	
Score 3	16 (41.0)	1 (2.6)	

*p* values are calculated by Fisher’s exact probability test. The ** corresponds to the significance levels at 1% (*p* < 0.01). SU-R, Shimane University postoperative hyphema scoring system-red blood cells score; TMH, Tanito microhook trabeculotomy; CE, cataract extraction.

**Table 6:** Preoperative and postoperative ophthalmologic variables.

Parameters	BCVA, LogMAR			ACF, pc/msec			CECD, Cells/mm^2^		
	TMH-CE	Inject-CE	*p*-Value	TMH-CE	Inject-CE	*p*-Value	TMH-CE	Inject-CE	*p*-Value
Preoperative value									
Mean ± SD	0.3 ± 0.3	0.2 ± 0.3	0.0003 **	9.1 ± 4.9	8.7 ± 6.2	0.30	2404.8 ± 288.5	2543.0 ± 251.0	0.0011 **
95% CI	0.2, 0.4	0.1, 0.3		7.5, 10.8	6.6, 10.9		2312.6, 2504.9	24611.0, 2624.4	
2 weeks postop									
Mean ± SD	0.3 ± 0.5	0.1 ± 0.2	0.0050 **	53.6 ± 88.0	31.0 ± 24.6	0.06			
95% CI	0.1, 0.4	0.0, 0.1		13.8, 83.4	22.7, 39.3				
1 month postop									
Mean ± SD	0.2 ± 0.4	0.0 ± 0.1	<0.0001 **	27.3 ± 20.9	19.2 ± 11.3	0.025 *			
95% CI	0.0, 0.3	−0.0, 0.0		19.0, 35.6	14.8, 23.7				
3 months postop									
Mean ± SD	0.1 ± 0.2	0.0 ± 0.1	0.07	18.8 ± 9.9	15.2 ± 11.6	0.09			
95% CI	0.0, 0.2	−0.0, 0.1		13.5, 24.0	9.0, 21.3				
6 months postop									
Mean ± SD	0.2 ± 0.5	−0.0 ± 0.1	0.045 *	13.0 ± 5.5	11.6 ± 5.9	0.22	2164.3 ± 393.8	2334.8 ± 330.0	0.021 *
95% CI	−0.0, 0.3	−0.0, 0.0		10.9, 15.1	9.5, 13.8		2024.7, 2304.0	2218.0, 2451.6	
9 months postop									
Mean ± SD	0.2 ± 0.5	−0.0 ± 0.1	0.010 *	11.6 ± 5.5	10.3 ± 4.7	0.15			
95% CI	−0.0, 0.4	−0.1, 0.0		9.5, 13.7	8.5, 12.1				
12 months postop									
Mean ± SD	0.2 ± 0.5	−0.0 ± 0.1	0.0044 **	9.7 ± 5.0	9.1 ± 4.0	0.44	2220.6 ± 310.2	2347.0 ± 281.3	0.06
95% CI	−0.0, 0.3	−0.1, 0.0		7.9, 11.6	7.7, 10.6		2212.4, 2328.8	2248.8, 2445.1	

Comparison between the TMH-CE and inject-CE groups using the Wilcoxon signed rank test. * *p* < 0.05, ** *p* < 0.01. BCVA, best-corrected visual acuity; LogMAR, logarithm of the minimum angle of resolution; ACF, anterior chamber flare; pc, photocount; msec, millisecond; CECD, corneal endothelial cell density; mm^2^, square millimeter; TMH, Tanito microhook trabeculotomy; postop, postoperatively; SD, standard deviation; CI, confidence interval.

**Table 7:** Postoperative changes in ophthalmologic variables.

Parameters	ΔBCVA, LogMAR			ΔACF, pc/msec			ΔCECD, Cells/mm^2^		
	TMH-CE	Inject-CE	*p*-Value	TMH-CE	Inject-CE	*p*-Value	TMH-CE	Inject-CE	*p*-Value
2 weeks postop									
Mean ± SD	−0.0 ± 0.4	−0.1 ± 0.4	0.0003 **	46.9 ± 90.0	20.4 ± 18.7	0.023 *			
95% CI	−0.1, 0.1	−0.2, 0.0		15.0, 78.9	13.7, 27.0				
1 month postop									
Mean ± SD	−0.1 ± 0.4	−0.2 ± 0.4	0.38	18.5 ± 22.2	10.1 ± 8.3	0.032 *			
95% CI	−0.2, 0.0	−0.3, −0.0		9.4, 27.7	6.6, 13.5				
3 months postop									
Mean ± SD	−0.2 ± 0.2	−0.1 ± 0.1	0.10	10.4 ± 8.5	7.8 ± 11.9	0.12			
95% CI	−0.3, −0.1	−0.2, −0.0		5.7, 15.1	1.2, 14.4				
6 months postop									
Mean ± SD	−0.1 ± 0.4	−0.2 ± 0.3	0.24	4.3 ± 4.5	2.8 ± 7.1	0.56	−263.7 ± 366.4	−202.0 ± 243.3	0.51
95% CI	−0.3, 0.0	−0.3, −0.1		2.5, 6.0	0.1, 5.5		−397.1, −129.3	−288.2, −115.7	
9 months postop									
Mean ± SD	−0.1 ± 0.5	−0.2 ± 0.4	0.33	2.3 ± 6.1	1.5 ± 7.2	0.73			
95% CI	−0.3, 0.1	−0.3, −0.1		−0.2, 4.8	−1.5, 4.4				
12 months postop									
Mean ± SD	−0.1 ± 0.5	−0.2 ± 0.3	0.13	0.6 ± 0.6	1.0 ± 5.7	0.83	−208.2 ± 281.1	−190.9 ± 193.0	0.97
95% CI	−0.3, 0.0	−0.3, −0.1		−1.8, 3.1	−1.1, 3.1		−309.6, −106.9	−258.3, −123.6	

Comparison between the TMH-CE and inject-CE groups using the Wilcoxon signed rank test. The * and ** correspond to the significance level at 5% (*p* < 0.05) and 1% (*p* < 0.01) for the Wilcoxon signed rank test. ΔBCVA, postoperative changes of best-corrected visual acuity; LogMAR, logarithm of the minimum angle of resolution; ΔACF, postoperative changes of anterior chamber flare; pc, photocount; msec, millisecond; ΔCECD, postoperative changes of corneal endothelial cell density; mm^2^, square millimeter; TMH, Tanito microhook trabeculotomy; postop, postoperatively; SD, standard deviation; CI, confidence interval.

## Data Availability

Data are fully available upon reasonable request to the corresponding authors.
